# The impact of head teacher praise and criticism on adolescent non-cognitive skills: Evidence from China

**DOI:** 10.3389/fpsyg.2022.1021032

**Published:** 2023-01-06

**Authors:** Xiaomei Ye, Qiran Wang, Yiming Pan

**Affiliations:** ^1^National School of Development, Peking University, Beijing, China; ^2^School of Education, Zhejiang International Studies University, Hangzhou, China; ^3^Shenzhen Foreign Languages School, Shenzhen, China

**Keywords:** head teacher, praise, criticism, adolescent, non-cognitive skills

## Abstract

**Introduction:**

Although the importance of teacher feedback has been confirmed by a great number of studies, the association of head teacher praise and criticism with adolescents’ non-cognitive skills still needs more deeper and more extensive research. Therefore, how to improve the non-cognitive skills of adolescents, especially those with disadvantaged family and economic backgrounds, has become a key concern in the field of educational practice.

**Methods:**

Based on CEPS data, this paper used panel regression and PSM-DID methods to analyze the impact of head teacher feedback on an adolescent’s non-cognitive skills measured by the big-five personality scale.

**Results:**

It found that praise from head teachers favorably influenced adolescents’ extraversion, agreeableness, openness, and conscientiousness, yet significantly mitigates their neuroticism. Meanwhile, the effect of criticism from head teachers is bi-facial: It made a positive effect on adolescents’ extraversion and openness but impaired their conscientiousness and neuroticism. As rural adolescents notably lag in their non-cognitive skills and are much less likely to be praised by head teachers compared to their urban peers, we estimate that when rural adolescents are frequently praised by their head teachers at the same level as urban students, their gap in extraversion, agreeableness, neuroticism, openness, and conscientiousness would be narrowed by 12.51%, 16.58%, 11.35%, 14.25%, and 24.29%. This finding has significant implications for head teacher teaching and adolescent well-being.

**Conclusions:**

The study examined the effects of head teacher praise and criticism on adolescent non-cognitive skills. The results showed that adolescents who were often praised by head teachers developed better non-cognitive skills. While the effect of head teacher criticism was two-sided: it enhances extraversion and openness as well as heightens neuroticism and corrupts conscientiousness. We further analyzed the urban-rural gap in non-cognitive skills and found that rural adolescents significantly lagged, and they have a lower possibility to be often praised by the head teacher, but a higher probability to be criticized. Through the PSM-DID quasi-experimental design, it was suggested that more head teacher praise can improve the non-cognitive skills among adolescents. When rural adolescents are estimated to receive the same amount of praise as urban adolescents, the disparities reduction in their non-cognitive skills can become possible. Our findings are of great significance to promote adolescent non-cognitive skills development and improve educational equity in urban and rural areas.

## 1. Introduction

In recent decades, non-cognitive skills have received growing attention as a catchall term for skills or traits that are not captured by cognitive skills assessment, it includes conscientiousness, agreeableness, social and emotional skills, etc. (Durlak et al., 2011). Evidence from economic and psychological research has highlighted the role of non-cognitive skills in students’ academic achievement, lifetime income, and well-being (Heckman et al., 2006; Chernyshenko et al., 2018). Adolescence is a critical period for individual development, and helping adolescents develop good non-cognitive skills will benefit them lifelong (Abbasi et al., 2022). The PISA and SSES International Survey Project implemented by the OECD both take adolescent students’ non-cognitive performance as important assessment indicators (Lee and Stankov, 2018). Therefore, how to improve the non-cognitive skills of adolescents has become a key concern in educational practice.

Since students spend most of their time a day at school, school-related factors are important for adolescents’ non-cognitive development. For example, junior high school students in China spend nearly 70% of their time every day in school (Xiang, 2017). The head teacher is a class organizer, leader, and educator in China’s education system. In addition to being responsible for subject teaching, he/she is also fully responsible for all aspects of a class of students’ thinking, learning, health, and life. For example, they often take on multiple responsibilities of managing daily class activities, paying attention to students’ study and psychological state, and communicating with parents and other teachers, which is similar to the homeroom teacher in Switzerland and many other countries (Mykletun and Mykletun, 1999; Baeriswyl et al., 2021). They have a longer contact time with students and give more feedback on student than other teachers (Munir et al., 2020). In psychology, a significant other is any person who has great importance to an individual’s life or well-being (Andersen and Thorpe, 2009). Head teachers and adolescents have lots of intensive everyday interactions, as compared to their parents and their friends, which might be significant for them (Tatar, 1998). Available data indicate that criticism and praise are two important feedback methods in teaching practice. Teacher praise can promote positive behavior and prevent negative behavior in the classroom (Stormont et al., 2007; Daniel et al., 2022), while teacher criticism often escalates challenging behavior (Longobardi et al., 2018). As one of the significant others for adolescents, head teacher praise and criticism may impact their students in multifaceted aspects. However, there are fewer studies about the effects of praise and criticism from head teachers on adolescents’ non-cognitive skills.

Non-cognitive skills cover a range of skills such as conscientiousness, perseverance, and teamwork (Paunonen and Ashton, 2001; Heckman and Kautz, 2012, 2013; Johnson, 2014). Studies have found that the development of non-cognitive skills in early childhood will sustainably affect students’ educational achievements, health, and lifetime income (Heckman et al., 2006). However, many studies found that the non-cognitive skills of rural adolescents are underdeveloped compared to urban adolescents, which consists of current findings (Zheng et al., 2021). This may result from the fact that rural adolescents are in a more disadvantageous family upbringing environment, and parents are poorly educated and they have less economic income, making it difficult to provide material and emotional support for adolescents (Becker and Luthar, 2002; Huang, 2018). There are some common scales for measuring non-cognitive skills such as the Rosenberg Self-Esteem Scale (Rosenberg, 1965), the Internal-External Scale (I-E Scale; Rotter, 1966), and the big-five personality scale (Goldberg, 1992, 1993; Johnson, 2014). The big-five personality models include extraversion, agreeableness, neuroticism, conscientiousness, and openness to experience, these five aspects can explain 75% of personality differences in most people and be widely used in the measurement of non-cognitive skills (Marsh et al., 2010; McCrae, 2011). Hundreds of empirical studies have proven that the big-five personality scale was reliably observed by raters and observers (McCrae et al., 2004) in many cultures (Schmitt et al., 2007), and it exerts an important influence on all aspects of life (Ozer and Benet-Martinez, 2006). However, the current measurement of adolescents’ non-cognitive skills is not fully performed but mostly focuses on students’ behaviors, such as truancy and disciplinary violations (Jackson, 2018), and the questionnaire response rate (Cheng and Zamarro, 2018). Therefore, using the big-five personality theory to measure the non-cognitive skills of adolescents has become a new perspective.

Teacher feedback of praise and criticism may help develop their adolescent students’ non-cognitive skills, especially head teachers who have more contact with adolescents. Praise is suggested to be used more in school settings (Caldarella et al., 2020). It conduces to the self-concept of adolescents and their behaviors, as well as the classroom environment (Wu et al., 2010). As regard self-image, more compliments to students enhance their sense of capability (Parsons et al., 1982; Worrall et al., 1983), improve their self-confidence, self-concept, and self-competence, so as to eventually enable students to reach their potential (Amemiya and Wang, 2018). Compared to the students who are commended less often, Students under such circumstances perceive themselves to be more hard-working and smarter (Pintrich and Blumenfeld, 1985; Spilt et al., 2016). Explicit Praise links to behavioral changes in students, especially their compliance with rules (Matheson and Shriver, 2005) and learning behaviors. It inspires the students to improve their behavior, be interested in the task at hand, and motivate them to actively accept the upcoming learning task (Hamre and Pianta, 2001; O’Connor and McCartney, 2007). In addition, when adolescents are encouraged to behave in ways that will elicit more praising statements in the future, problem behaviors are expected to reduce saliently or be prevented (Howell et al., 2014).

On the contrary, criticism is commonly used when teachers giving negative feedback. In contrast, praise clobbers adolescents’ self-concept and worthwhileness (Doumen et al., 2011; Spilt et al., 2016; Weidinger et al., 2016). Students will usually be more reluctant or resistant to change their inappropriate behaviors, and even remain unwanted and disruptive participants in class if they are habitually or constantly exposed to reprimand (Gable et al., 2009; Spilt et al., 2016). According to recent research, students in China are more sensitive to negative feedback from teachers than those from the rest of the world, they will tend to undermine their abilities because of it (Li, 2022). Sometimes students will experience defense mechanism activation and turn to resentment and hatred (Diller, 2018). Over time, they tend to develop a deep-seated doubt in their self-worth even when they are praised or approved and disbelief in teachers’ concern for them (Spilt et al., 2016). Inattention, inaction or disordered behaviors, and mental illness in the worst case are often found among criticized students (Zhao, 2021). Given the nature of the nonspecific statement, for example, “Do not do that,” it fails to provide guidance or point out a more appropriate way for a student to behave, but instead aggregates low motivation and also is detrimental to their interest in developing academic skills and knowledge (Spilt et al., 2016; Weidinger et al., 2016). Thus, the consistent use of negative feedback impairs students’ self-perception and multifaceted abilities in the long run (Hamre and Pianta, 2001; Rose et al., 2012).

Unlike previous studies that focused on how teachers use feedback and how teachers’ feedback influences students’ learning ability, social behavior, and mental well-being (Salili and Hau, 1999; Wullschleger et al., 2020; Thompson et al., 2020), this study assessed the impact of head teachers’ praise and criticism on adolescents’ overall non-cognitive development. Since most studies have found that teacher praise can promote students’ academic success (Hamre and Pianta, 2001; O’Connor and McCartney, 2007; Moore et al., 2019) and teacher criticism can easily lead to negative behavior and mental health condition in students (Hamre and Pianta, 2001; Rose et al., 2012), this paper proposes the following two research hypotheses from the perspective of head teacher praise and criticism:

*H1*: Adolescents who are often praised by head teachers have significantly higher non-cognitive skills.

*H2*: Adolescents who are often criticized by head teachers have significantly lower non-cognitive skills.

Further, as many studies have found that the non-cognitive skills of rural students are less well-connected than that of urban students (Zheng et al., 2021), improving rural students’ outcome and bridging the urban–rural divide to achieve equity become a worldwide cause (Lounkaew, 2013). Since teacher praise mostly positively relates to students’ educational performance, this paper will explore whether the increasing use of head teacher praise to rural adolescents can achieve the balanced development of non-cognitive skills between urban and rural students. We hope to shed light on the rural–urban educational disparities and provide further evidence to support policy intervention on urban–rural education equity. This leads to the third research hypothesis:

*H3*: If other conditions remain unchanged, and rural and urban adolescents are praised by their head teachers at the same frequency, the gap in non-cognitive skills development between them would be narrowed.

## 2. Materials and methods

### 2.1. Participants

The data came from the China Education Panel Survey (CEPS), which aims to reveal the impact of family, school, community, and the macro social structure on individual educational outcomes. Two follow-up surveys were also conducted by the National Survey Research Center at Renmin University in China. In the first survey in the 2013–2014 school year, 19,847 students in the 7th and 9th grades were recruited from 438 classes in 112 schools in China. In the second survey in the 2014–2015 school year, the follow-up success rate was over 90%. The database includes information on non-cognitive skills in adolescence, perception of head teacher praise and criticism, demographic characteristics, family background, and other information that supports this research. Finally, we selected 8,400 adolescents in the 7th grade during the first survey and follow-up the second survey, including 3,984 urban and 4,416 rural adolescents, among them 4,284 boys and 4,116 girls.

### 2.2. Measures

#### 2.2.1. Non-cognitive skills in adolescence

The dependent variables were the non-cognitive skills of adolescents. We measured them based on the CEPS questionnaire using the big-five personality model, including conscientiousness, extraversion, openness to experience (namely openness), agreeableness, and neuroticism. The five indicators in the paper referred to the definition of the big-five personalities provided by the American Psychological Association and the M5-120 scale. And the M5-120 scale was internationally used and verified by hundreds of empirical studies for non-cognitive skills measurements (Johnson, 2014; Bastian et al., 2017). Extroversion refers to the tendency to be enthusiastic, social, proactive, and optimistic, as in “I often participate in activities organized by the school or class.” Agreeableness means personal qualities such as altruism, trust, cooperation, empathy, etc., and is measured by three items (Most of my classmates are friendly to me; The class I am in has a good atmosphere; and I feel close to the people at my school). Conscientiousness suggests personal qualities such as competence, responsibility, self-discipline, diligence, and self-efficacy, and is measured by four items (Even if I am a little unwell or have other reasons to stay home, I still try to go to school; Even if it is the homework I do not like, I try my best to do it; Even if it takes a long time to complete the homework, I will continue to try my best to do it; and I am confident in my future). Neuroticism indicates anxiety, depression, vulnerability, and other negative emotions, it is measured by six items (In the last seven days, I have felt depressed, blue, unhappy, bored, and sad; I was bored at school). Openness, meaning having a wide range of emotions, being creative, intelligent, and innovative, is measured by five items (I can express my opinion clearly; My reflexes are swift; I can learn new knowledge quickly; I am curious about new things; and I hope to go to a different school to study). Each item has five options: Strongly disagree, disagree, neutral, agree, and strongly agree, assigned a value of 1–5. The reliability and validity tests of these items showed that the KMO was 0.868 and the Cronbach’s alpha was 0.856. This paper averaged the relevant items of each dimension and standardized these five indicators.

#### 2.2.2. Head teacher praise and criticism

The core explanatory variable we focused on was head teacher praise and criticism perceived by adolescents, such feedback could often change their mental models and behaviors. Therefore, this paper measured the head teacher’s praise and criticism by using self-assessments, asking, “My head teacher always praises me,” and “My head teacher always criticizes me.” And adolescents chose four items from “totally disagree” to “totally agree.” According to the sample distribution, we counted the values of “totally disagree and relatively disagree” to “the head teacher does not praise/criticize me often” as 0, and those of “comparatively agree and totally disagree” to “the head teacher often praises/criticizes me” as 1. Higher values represented more praise/criticism given by the head teacher.

#### 2.2.3. Students’ background questionnaire

Considering that non-cognitive skills in adolescence are also influenced by factors such as individual characteristics and family background, we controlled the following variables in the estimation: gender (girl = 0, boy = 1), birth of year (in years), rural residence (rural = 1, urban = 0), living with parents (yes = 1, no = 0), kindergarten experience (yes = 1, no = 0), the only child in family (yes = 1, no = 0), family economic status (poor = 1, average = 2, rich = 3), boarding at school (Do you board at school from Monday to Friday? yes = 1, no = 0), parents’ educational expectations for their children, mother’s education level, and father’s education level. The last three variables about academic level scored from 1 (not educated) to 9 (postgraduate degree or above). Table 1 reports the descriptive statistics.

**Table 1:** Urban–rural adolescents’ difference in non-cognitive abilities and head teachers’ feedback.

	Adolescent non-cognitive skills					Head teacher feedback	
	Extraversion	Agreeableness	Neuroticism	Openness	Conscientiousness	Praise	Criticism
Urban students	0.131	0.128	−0.125	0.063	0.161	0.478	0.151
	(0.969)	(0.975)	(1.002)	(0.984)	(0.977)	(0.500)	(0.358)
Rural students	−0.052	−0.032	−0.036	−0.039	0.093	0.432	0.175
	(1.008)	(0.998)	(0.939)	(0.959)	(0.933)	(0.495)	(0.380)
*t*-test	0.182***	0.159***	−0.090***	0.102***	0.068***	0.046***	−0.024***

1. Value in parentheses is standard error. 2. *p < 0.05, **p < 0.01, ***p < 0.001.

### 2.3. Identification strategy

In order to estimate the impact of head teacher praise and criticism on adolescents’ non-cognitive skills, we used the education production function model. To reduce estimation bias we used two periods of panel data from 2014 and 2015. Panel data can reflect the dynamic development of adolescents’ non-cognitive skills and account for the interference of some variables (such as natural maturity, living environment, etc.) that do not change over time and, in doing so, obtain more effective causal inference. Therefore, we established a panel data regression model, as shown in Equation (1):


(1) $$Y_{it}=\alpha +\beta TP_{it}+\gamma TC_{it}+\delta P_{it}+\theta F_{it}+\varepsilon_{it}$$

In Equation (1), *t* means the year of observation (2014 or 2015), $Y_{it}$ is the non-cognitive skills (including extraversion, agreeableness, conscientiousness, openness, and neuroticism) of adolescent *i* at time point *t*. $TP_{it}$ is the frequency of their head teacher praise (often or rarely) perceived by adolescents *i*. $TC_{it}$ means the frequency of their head teacher criticism (often or rarely) perceived by adolescents *i*. $P_{it}$ indicates the control variable for adolescents *i*, accounting for gender, year of birth, residence, hukou (Household Registration), whether the child lives with parents, whether the child has attended kindergarten, whether the child has siblings, etc. $F_{it}$ refers to the family background of adolescents *i*, accounting for family economic background, parents’ education level, parents’ expectation on children’s education etc. $\varepsilon_{it}$ is the error item.

In order to provide accurate results for potential policy improvement, this paper further used the quasi-experimental method of PSM-DID to estimate the impact of the changes in head teacher feedback. To compare the differences in non-cognitive skills between the test group who had head teacher feedback changed from 2014 to 2015 and the control group who had head teachers’ feedback stayed the same, it is necessary to ensure that characteristics of adolescents in the control and treatment group are similar. Within the control and treatment groups, there was the group with head teacher praise increasing and the group with praise not changing, the group with head teachers’ praise decreasing and the group with praise not changing, the group with head teachers’ criticism increasing and the group with criticism not changing, the group with head teacher criticism decreasing, and the group with criticism not changing. We used the logit model to calculate the probability of the adolescents get praised or criticized by their head teacher in the control group and the treatment group. The adolescent and family variables were used as the vector *M* for calculation.

In Equation (2) $,\theta_{1}$ is a constant term, $\;\theta_{2}$ is the coefficient matrix of vector *M*.


(2) $$\ln\left(\frac{p}{1-p}\right)=\theta_{0}+\theta_{1}M$$

After obtaining the samples of the control and treatment groups, we constructed the following difference in difference model.


(3) $$\begin{array}{l} Y_{it}=\alpha_{0}+\alpha_{1}Treat_{itn}+\alpha_{2}year+\alpha_{3}Treat_{itn}\\ \times year+\alpha_{4}M+\xi_{it} \end{array}$$

In Equation (3) *n* denotes the type of treatment: *n* = 1 denotes the increase of the head teacher praise, *n* = 2 denotes the decrease of the head teacher praise, *n* = 3 denotes the increase of the head teacher criticism, and *n* = 4 denotes the decrease of the head teacher criticism. *Year* is a dummy variable, *year* = 1 means the current school year, and *year* = 0 means before the current school year. $\;\alpha_{0}$ is a constant term, *ξ* is the residual term. The Coefficient $\alpha_{3}$ of the *Treat* × *year* interaction term is the effect of change of head teacher feedback on the adolescents’ non-cognitive skills.

## 3. Results

### 3.1. Adolescents’ non-cognitive skills and head teacher praise and criticism

Table 1 shows that rural adolescents’ non-cognitive skills were significantly inferior to urban adolescents, especially in extraversion, agreeability, openness, and conscientiousness. In contrast, rural adolescents score much higher than urban adolescents in neuroticism. It also shows that urban adolescents are likely to be more praised and less criticized by head teachers than rural peers. The results of the *t*-test of these indicators show significant differences between urban and rural students, all significant at the 0.01 level.

### 3.2. The impact of head teacher praise and criticism on adolescents’ non-cognitive skills

Based on the Model in Equation (1), the panel regression method is used to estimate the effect of head teacher praise and criticism on adolescent non-cognitive skills. Model (1)–(5) in Table 2 shows that head teacher praise can significantly improve adolescents’ non-cognitive skills, including extraversion, agreeableness, openness, and conscientiousness by 0.469, 0.534, 0.309, and 0.355 standard deviations while reducing their neuroticism by 0.222 standard deviations. All of these estimated coefficients were significant at the level of 0.01. Model (6)–(10) in panel B shows that head teacher praise contributed to higher extraversion, agreeableness, openness, and conscientiousness but also lower neuroticism among urban adolescents. Furthermore, Model (11)–(15) in panel C shows that rural adolescents’ non-cognitive skills benefit from more head teacher praise. These results indicate that when adolescents perceive that their head teacher often praises them, their non-cognitive skills develop better, which is consistent among both urban and rural adolescent groups.

**Table 2:** Impact of head teacher praise and criticism on adolescents’ non-cognitive skills.

Panel A: Total	Extraversion	Agreeableness	Conscientiousness	Neuroticism	Openness
	(1)	(2)	(3)	(4)	(5)
Praise	0.469***	0.534***	0.355***	−0.222***	0.309***
	(0.015)	(0.014)	(0.015)	(0.015)	(0.015)
Criticism	0.094***	−0.001	−0.140***	0.284***	0.059***
	(0.019)	(0.019)	(0.019)	(0.019)	(0.020)
Control variables	√	√	√	√	√
Observation	16,800	16,800	16,800	16,800	16,800
Number of ID	8,400	8,400	8,400	8,400	8,400
**Panel B: Urban**	**(6)**	**(7)**	**(8)**	**(9)**	**(10)**
Praise	0.442***	0.488***	0.348***	−0.220***	0.301***
	(0.021)	(0.021)	(0.022)	(0.022)	(0.022)
Criticism	0.077***	−0.023	−0.161***	0.291***	0.063**
	(0.028)	(0.028)	(0.030)	(0.029)	(0.031)
Control Variables	√	√	√	√	√
Observation	7,968	7,968	7,968	7,968	7,968
Number of ID	3,984	3,984	3,984	3,984	3,984
**Panel C: Rural**	**(11)**	**(12)**	**(13)**	**(14)**	**(15)**
Praise	0.495***	0.573***	0.359***	−0.222***	0.316***
	(0.021)	(0.020)	(0.020)	(0.020)	(0.021)
Criticism	0.106***	0.016	−0.125***	0.277***	0.055**
	(0.027)	(0.026)	(0.025)	(0.025)	(0.027)
Control Variables	√	√	√	√	√
Observation	8,832	8,832	8,832	8,832	8,832
Number of ID	4,416	4,416	4,416	4,416	4,416

1. Value in parentheses is standard error. 2. *p < 0.05, **p < 0.01, ***p < 0.001.

It also found that head teachers’ criticism has both positive and negative effects on adolescent non-cognitive skills. For example, when the head teacher regularly criticizes adolescents, their extraversion and openness could increase by 0.094 and 0.059 standard deviations, respectively. However, their conscientiousness would reduce by 0.140 standard deviations, and neuroticism would increase by 0.284 standard deviations. All of these estimated coefficients were significant at the level of 0.01. Model (6)–(10) in panel B shows that head teacher criticism positively affected urban adolescents’ extraversion, neuroticism, and openness while negatively affecting their conscientiousness. Furthermore, Model (11)–(15) in panel C shows the effect of head teacher criticism on rural adolescents’ non-cognitive skills, which is similar to the impact on urban adolescents.

### 3.3. Robustness test

We used the quasi-experimental method of PSM-DID to estimate the impact of changes in the frequency of head teacher feedback on adolescents’ non-cognitive skills. There are four types of frequency changes that we based on to set up four treatment groups and control groups: an increase in the frequency of praise, a decrease in the frequency of praise, an increase in the frequency of criticism, and a decrease in the frequency of criticism. In order to ensure the similar background characteristics of the two groups, we used the propensity matching scores (PSM) to estimate the sample size of each pair of treatment and control groups. Table 3 shows that before using PSM, substantial differences occurred between the treatment group and the control group in each experiment, but they became less obvious after matching.

**Table 3:** Impact of head teacher praise and criticism on adolescents’ non-cognitive skills (PSM-DID).

	Extraversion	Agreeableness	Conscientiousness	Neuroticism	Openness
Increase praise	0.385***	0.482***	0.166***	−0.025	0.299***
	(0.048)	(0.048)	(0.046)	(0.048)	(0.047)
Control variables	√	√	√	√	√
Treatment group	1,148	1,148	1,148	1,148	1,148
Control group	3,101	3,101	3,101	3,101	3,101
Difference before matching	29.94***	29.94***	29.94***	29.94***	29.94***
Difference after matching	3.04	3.04	3.04	3.04	3.04
Decrease praise	−0.286***	−0.312***	−0.246***	0.063	−0.297***
	(0.040)	(0.038)	(0.040)	(0.041)	(0.042)
Control Variables	√	√	√	√	√
Treatment group	1,759	1,759	1,759	1,759	1,759
Control group	2,327	2,327	2,327	2,327	2,327
Difference before matching	55.44***	55.44***	55.44***	55.44***	55.44***
Difference after matching	3.34	3.34	3.34	3.34	3.34
Increase criticism	0.235***	0.185***	0.009	0.166***	0.041
	(0.044)	(0.044)	(0.042)	(0.042)	(0.043)
Characters control	√	√	√	√	√
Treatment group	1,161	1,161	1,161	1,161	1,161
Control group	6,030	6,030	6,030	6,030	6,030
Difference before matching	166.17***	166.17***	166.17***	166.17***	166.17***
Difference after matching	6.77	6.77	6.77	6.77	6.77
Decrease criticism	0.011	−0.026	0.050	−0.121	−0.206**
	(0.086)	(0.086)	(0.088)	(0.093)	(0.094)
Characters control	√	√	√	√	√
Treatment group	745	745	745	745	745
Control group	408	408	408	408	408
Difference before matching	21.26**	21.26**	21.26**	21.26**	21.26**
Difference after matching	1.56	1.56	1.56	1.56	1.56

1. Value in parentheses is standard error. 2. *p < 0.05, **p < 0.01, ***p < 0.001.

The first experiment was to estimate the influence of an increase in the frequency of head teacher praise. The treatment group (*n* = 1,148) was the adolescents who had rarely been praised by their head teacher in the baseline survey and were frequently praised in the follow-up investigation the next year, and the control group (*n* = 3,101) was the adolescents who were rarely praised by their head teacher both in baseline and follow-up surveys. The second experiment was to estimate the impact of a decrease in the frequency of head teacher praise, in which the treatment group (*n* = 1,759) was the adolescents who had often been praised by their head teacher in the baseline survey but were rarely praised in the follow-up investigation after 1 year, and the control group (*n* = 2,327) was adolescents who were frequently praised by their head teacher both in baseline and follow-up surveys. The third experiment was to estimate the impact of an increase in the frequency of head teacher criticism, in which the treatment group (*n* = 1,161) was the adolescents who had seldom been criticized by their head teacher in the baseline survey and were frequently criticized in the follow-up investigation 1 year later, and the control group (*n* = 6,030) was the adolescents who were seldom criticized both in both surveys. The fourth experiment was to estimate the impact of a decrease in the frequency of head teacher criticism, in which the treatment group (*n* = 745) was the adolescents who had often been criticized by their head teacher in the baseline survey but were rarely criticized in the follow-up investigation after 1 year, and the control group (*n* = 408) was the adolescents who were often criticized both in the first and second observations.

Table 3 shows that an increase in the frequency of praise by head teachers can considerably progress extraversion, agreeableness, openness, and conscientiousness, with coefficients of 0.385, 0.482, 0.299, and 0.166, respectively. While a decrease in the frequency of head teacher praise represses extraversion, agreeableness, openness, and conscientiousness, with coefficients of −0.286, −0.312, −0.29, and −0.246 respectively, all of them are significant at 1%. The results suggest that the positive non-cognitive skills will become better when adolescents get more praise from their head teacher. The praise of head teacher rarely will hinder the development of some positive non-cognitive skills.

On the other hand, the increase in head teacher criticism had a significant effect on extraversion, agreeableness, and neuroticism which had coefficients of 0.235, 0.185, and 0.166 respectively, all of them being significant at 1%. And the decrease in the frequency of head teacher criticism significantly reduces adolescents’ openness with a coefficient of −0.206 (*p* < 0.01). This implies that the increase in head teacher criticism has a two-sided effect: it can promote extraversion and agreeableness, but also incite neuroticism. Therefore, head teachers should be mindful of using criticism during daily educational management and pay attention to their students’ mental well-being.

### 3.4. Assessment of narrowing the gap between urban and rural adolescents

As shown in Table 1, rural adolescents have lower non-cognitive skills than urban peers, and they are less frequently praised and more frequently criticized by head teachers. Table 3 shows that the increase in head teacher praise can greatly enhance adolescents’ non-cognitive skills, while the decrease in criticism has little effect. We assume that the gap between rural and urban adolescents’ non-cognitive skills will be narrowed if they receive the same amount of praise from head teachers, and the effect of increasing head teacher praise is estimated by referring to the method of Magnuson and Waldfogel (2010). Table 4 shows that if the frequency of praise to rural adolescents is raised to the level as urban adolescents, the gap in non-cognitive skills between them will be narrowed. This means when the frequency of head teacher praise in rural areas rises from 43.2 to 47.8%, the gap in extraversion between urban and rural adolescents is lowered by 12.51%, the gap in agreeableness is reduced by 16.58%, the gap in neuroticism drops by 11.35%, the gap in openness is diminished by 14.25%, and the gap in conscientiousness is reduced by 24.29%.

**Table 4:** Simulated results for increasing head teacher praise of rural adolescents.

Increase the frequency of head teacher praise of rural adolescents to that of urban 0.432 → 0.478 ①	The effect of head teacher praise on rural students (②)	Overall average growth (③ = ① * ②)	Contribution to narrowing the gap of non-cognitive skills (④ = ③/gap of non-cognitive skills)
Extraversion	0.495	0.023	12.51%
Agreeableness	0.573	0.026	16.58%
Neuroticism	−0.222	−0.010	11.35%
Openness	0.316	0.015	14.25%
Conscientiousness	0.359	0.017	24.29%

1. Statistics in the first column is from Table 1 that represents when rural adolescents have the same frequency of head teacher praise as urban adolescents. 2. The effect of head teacher praise shown in the third column comes from the OLS regression coefficient in Panel B of Table 3.

## 4. Discussion

Although previous studies have examined the effects of teacher feedback on students’ development, empirical evidence on its effects from the head teacher on adolescents’ non-cognitive skills still needs to be sparse. As non-cognitive skills are a vital component of adolescent development, significantly impacting their educational achievement, future labor market incomes, and well-being, improving students’ non-cognitive skills was highly emphasized by the whole region in recent years. On a more fundamental level, head teachers are important others in the growth of adolescents, their influence on adolescents is more significant than that of other teachers while less researched. This paper used the China Education Panel Survey (CEPS) data, and adopted panel regression and PSM-DID methods to examine the impact of head teacher praise and criticism on adolescents’ non-cognitive skills. The result enriches head teachers’ research and provides empirical evidence for head teachers to implement effective feedback strategies. In addition, educational equity between urban and rural areas has been a critical issue internationally while less researched from the perspective of head teacher feedback. We further examined heterogeneity between urban and rural adolescents, and used policy simulations to estimate the contribution of increased head teacher praise to narrow the urban–rural gap of adolescent non-cognitive skills. The result is of great significance to formulating education equity policy and promoting the development of disadvantaged rural adolescents.

### 4.1. The influence of head teacher praise on adolescents’ non-cognitive skills

Research has revealed that teacher praise can affect students’ academic success, such as their positive study behavior and performance would be affected by teacher feedback in different ways (Moore et al., 2019; Wullschleger et al., 2020). However, previous studies have ignored bringing non-cognitive skills into the scope of the investigation. Consistent with the first hypothesis, this paper found that adolescents who are often praised by head teachers develop better non-cognitive skills. Specifically, the extraversion, agreeableness, conscientiousness, and openness of adolescents who are often praised by the head teacher will be higher than those who receive less praise by 0.469, 0.534, 0.355, and 0.309 standard deviations, and their neuroticism would be lowered by −0.222 standard deviations. In general, non-cognitive skills in four dimensions, except neuroticism, are positively correlated with a student’s educational achievement (Johnson, 2014; Jackson, 2018). Therefore, the head teacher’s praise can significantly promote the development of non-cognitive skills in adolescents, which shows consistency with the estimations of urban and rural student groups. It is likely because teacher praise can significantly motivate students to learn and improve their inner characters (Liu et al., 2015; Guo and Wei, 2019), as well as regulate emotions (Caldarella et al., 2019). Additionally, based on the theory of “significant others,” research had found that interactive behaviors from teachers could influence students in many ways (Vervoort et al., 2014). Head teacher is a major factor in adolescence, he or she has the closest ties with students and also makes difference in their learning, school life, class management (Forde et al., 2022) as well as their overall life development (Xie et al., 2021). In line with the first hypothesis, praise from the head teacher could help adolescents develop in many aspects, especially in non-cognitive skills. The result reveals dual reasons for the positive changes a head teacher can bring to adolescents’ non-cognitive skills: the effect of praise and the importance of a head teacher.

### 4.2. The influence of head teacher criticism on adolescents’ non-cognitive skills

Although previous studies appear that overmuch criticism from teachers would reduce students’ learning motivation and enthusiasm (Spilt et al., 2016; Weidinger et al., 2016), they do not consider how criticism affected adolescents’ non-cognitive skills or whether it could be a two-way influence. Not entirely consistent with the second research hypothesis, this paper finds that head teacher criticism serves both positive and negative results. Adolescents who are often criticized by the head teacher have higher extraversion and openness than those who are less criticized by 0.094 and 0.059 standard deviations, but their neuroticism rises by 0.284 standard deviations and conscientiousness is significantly lowered by −0.140 standard deviations. Such tendencies occurred in both urban and rural student groups. The reason behind this might be that criticism is often perceived as a social threat, and constant exposure to head teacher criticism may form a negative self-image as students grow up and even cause mental problems (Harris and Howard, 1984). Contrary to existing research (Van Houtum et al., 2022), this paper found that adolescents who are often criticized by head teachers show significantly higher levels of extraversion and open-ended development. It may be explained that criticism even as a form of negative feedback still increases the frequency of interaction between teachers and students. When a head teacher uses criticism effectively, adolescents feel that “criticism is also caring” and accordingly develop healthy personalities.

### 4.3. The contribution of head teacher praise to narrowing the rural–urban gap of adolescents’ non-cognitive skills

Furthermore, through the quasi-experimental design of PSM-DID, we discovered that the increase in head teacher praise can significantly promote the non-cognitive skills of adolescents. As rural adolescents lagged significantly behind in non-cognitive skills development and they are praised by head teachers less often. This paper evaluates the contribution of raising the frequency of head teacher praise to rural adolescents toward the urban average level to narrow the gap in their non-cognitive skills. The results show that after the probability of rural adolescents being often praised by the head teacher was increased from 43.2% to 47.8%, the extraversion, agreeability, neuroticism, openness, and conscientiousness of urban and rural adolescents would be reduced by 12.51%, 16.58%, 11.35%, 14.25%, and 24.29%. This validates the third research hypothesis in this paper. Due to the disadvantaged economic and cultural status of rural families, the lower level of non-cognitive skills of rural adolescents will impede realizing educational equity in urban and rural areas (Gu and Yeung, 2020). Seeking factors in the school setting as opposed to in a family setting is more plausible to find solutions. The evaluation of policy intervention in this paper also shows that increasing the praise of head teachers can narrow the gap in non-cognitive skills between urban and rural adolescents.

The paper also shows that both praise and criticism from head teachers can have an important impact on the non-cognitive skills of adolescents, especially the praise, which can promote the non-cognitive skills of adolescents but also narrow the gap between urban and rural. The findings are consistent with previous research results that teacher praise plays a positive and important role in teaching practice (Zhang et al., 2021). However, influenced by the Chinese traditional educational idea that “An accomplished student owes his accomplishment to his strict teacher,” most teachers still use “More criticism and less praise” in the process of teaching (Xie et al., 2021). Another factor is that some teachers in China are lacking critical thinking skills when giving feedback to students (Azid et al., 2020). In terms of the policies in China to improve teacher quality and student development, we attach importance to the role that teacher praise can play. Besides, in-service training can help head teachers better understand the effect of praise and criticism and then master effective feedback methods (Baocun et al., 2015; Bjørndal, 2022). Non-cognitive skills will be developed when more effective praise and less ineffective criticism are used in teaching. Adolescents are at an important stage of developing non-cognitive skills, the sooner they encounter these interventions, the greater the benefits they will receive (Heckman et al., 2010). This paper provides empirical evidence to support intervention policies regarding head teacher feedback to improve the non-cognitive skills of urban and rural adolescents, as well as to bridge the gap of non-cognitive skills between urban and rural adolescents, namely education equity. This research provides a valuable reference for instructing teachers on adopting the proper feedback and promoting educational equity.

## 5. Limitations

The study provides a new perspective on improving non-cognitive skills in adolescence and promoting urban–rural education equity by exploring the impact of head teacher criticism and praise. Despite the contributions of our research, some limitations should be mentioned. First, due to data limitations, we measured adolescents’ non-cognitive skills based on the existing research and evaluation information, so some data used may not be what was needed. Second, the feedback of praise and criticism in the study was reported from the perspective of adolescents rather than from their head teachers. Moreover, only a single item was used to measure the praise/criticism of the head teacher. In the following research, we can collect data from head teachers to see if there is a cognition difference between students and head teachers. We hope to understand how teachers view the means of giving more praise and less criticism. More research is needed in the future to comprehensively assess the relationship between Chinese adolescents’ non-cognitive skills and head teacher praise and criticism.

## 6. Conclusion

The study examined the effects of head teacher praise and criticism on adolescent non-cognitive skills. The results showed that adolescents who were often praised by head teachers developed better non-cognitive skills. While the effect of head teacher criticism was two-sided: it enhances extraversion and openness as well as heightens neuroticism and corrupts conscientiousness. We further analyzed the urban–rural gap in non-cognitive skills and found that rural adolescents significantly lagged, and they have a lower possibility to be often praised by the head teacher, but a higher probability to be criticized. Through the PSM-DID quasi-experimental design, it was suggested that more head teacher praise can improve the non-cognitive skills among adolescents. When rural adolescents are estimated to receive the same amount of praise as urban adolescents, the disparities reduction in their non-cognitive skills can become possible. Our findings are of great significance to promote adolescent non-cognitive skills development and improve educational equity in urban and rural areas.

## Data availability statement

Publicly available datasets were analyzed in this study. These data can be found at: http://ceps.ruc.edu.cn/.

## Author contributions

XY is responsible for the design, data analysis, and related discussions of the article. QW is responsible for the design of the article, literature review, and related discussions. YP is responsible for the modification and revision of the article. All authors contributed to the article and approved the submitted version.

## Conflict of interest

The authors declare that the research was conducted in the absence of any commercial or financial relationships that could be construed as a potential conflict of interest.

## Publisher’s note

All claims expressed in this article are solely those of the authors and do not necessarily represent those of their affiliated organizations, or those of the publisher, the editors and the reviewers. Any product that may be evaluated in this article, or claim that may be made by its manufacturer, is not guaranteed or endorsed by the publisher.
